# DNA extraction for human microbiome studies: the issue of standardization

**DOI:** 10.1186/s13059-019-1843-8

**Published:** 2019-10-21

**Authors:** K. Leigh Greathouse, Rashmi Sinha, Emily Vogtmann

**Affiliations:** 1 Nutrition Sciences, Robbins College of Health and Human Sciences, Baylor University, Waco, TX USA; 2 Metabolic Epidemiology Branch, Division of Cancer Epidemiology & Genetics, National Cancer Institute, National Institutes of Health, Bethesda, MD USA

## Abstract

Among the laboratory and bioinformatic processing steps for human microbiome studies, a lack of consistency in DNA extraction methodologies is hindering the ability to compare results between studies and sometimes leading to errant conclusions. The purpose of this article is to highlight the issues related to DNA extraction methods and to suggest minimum standard requirements that should be followed to ensure consistency and reproducibility.

## Introduction

Standardization in microbiome analyses is fundamental to reliably measure the human microbiome. Variability in results can stem from all the steps in the human microbiome study process including sample collection, DNA extraction, sequencing, and bioinformatics. Of these, DNA extraction was identified by the MicroBiome Quality Control project (MBQC) [1], the International Human Microbiome Standards (IHMS) group [2], and others as contributing a majority of experimental variability. Thus, while each aforementioned aspect is important, in this commentary, we will only cover issues related to DNA extraction from different human sample types and the inclusion of appropriate reference material for human microbiome research.

## DNA extraction from fecal samples

A variety of protocols are available to extract microbial DNA from human samples, but the literature has predominantly focused on protocols for extraction from fecal samples. The Human Microbiome Project (HMP) [3], MetaHIT [4], and the Earth Microbiome Project [5] and many other groups have published DNA extraction protocols for feces, as well as, other sample types.

Both the MBQC and IHMS evaluated the impact of different DNA extraction protocols for fecal samples, and they found that DNA extraction protocols had the largest impact on experimental variability [1, 2]. The source of variability between DNA extraction methods could be related to multiple factors including reagent contamination, type of lysis (e.g., mechanical or enzymatic), differences between laboratory personnel or automation of DNA extraction, among many other factors. The IHMS study also proposed a DNA extraction protocol that maximized ease of use and reproducibility [2], although currently this method has not been automated. In a study directly comparing the HMP and MetaHIT extraction methods for fecal samples, the MetaHIT protocol yielded a higher number of read mapping to eukaryotic genomes while the HMP protocol had a greater number of reads mapping to bacterial genomes. Even within the analysis of bacteria, the two methods detected differing abundances of specific genera [6]. These studies indicate that a standardized protocol for DNA extraction from fecal samples may be ideal to decrease variability due to DNA extraction, although using standardized protocols will not prevent other inter-laboratory differences. Even though multiple potential standard protocols exist for fecal samples, the selection of a standardized protocol for all studies is still extremely difficult due to many factors, including a need for automation of DNA extraction methods for large sample sizes and a lack of understanding of the true composition of a fecal sample to serve as the ground truth for comparisons between methods. Inclusion of quality control samples as discussed below would help address some of these issues, but more studies to evaluate DNA extraction protocols for fecal samples are needed.

While some standard DNA extraction methods have been proposed for fecal samples, these methods are not always appropriate for other human sample types. For example, oral samples contain higher concentrations of human DNA compared to fecal samples [7, 8], which can affect DNA extraction and downstream procedures. Methods are available to deplete host DNA during extraction [9]; however, these methods are quite costly and may also bias DNA extraction and affect the resulting microbial community. Overall, while protocols for DNA extraction have been studied for fecal samples, care should be taken when adapting these methods for other sample types, especially non-fecal and low-biomass samples.

## DNA extraction from samples with low microbial biomass

Several human microbiome studies have also focused on samples with lower microbial biomass, such as human tissue samples and spinal and joint fluids. While samples with high microbial DNA biomass, such as fecal samples, are less subject to biases or false positives due to contamination during processing, low biomass samples are highly subject to contamination from exogenous sources [10, 11]. Two complementary methods are required to avoid most but not all significant errant results: (1) reduction of possible contaminants and (2) demonstration of the existence of microbial life beyond sequencing.

As it relates to contamination in low biomass samples, several previous studies have identified common contaminants emanating from DNA extraction kits and other laboratory reagents [11, 12], but beyond this list, researchers must carefully evaluate all possible sources of contamination at each stage of the study, ranging from sample collection to laboratory processing. As an example, cancer-associated microbiome studies using low biomass tissue such as lung tissue should start by including numerous samples of possible contamination and minimizing contamination at the source. In this scenario, one would begin with the origin of the sample tissue collection location, identify all possible environmental sources of contamination, and collect swabs of air, surgical tools, gloves, collection tubes, patient skin, and other samples, until all points of contacts have been identified for this tissue being collected immediately prior to storage or freezing. It is also ideal to collect low biomass samples in an environment designed to minimize as much contamination as possible. This can be established, for example, by creating a sterile pathology suite next to the operating room that is optimized for microbiome studies, including staff trained in sterile techniques. Specific protocols for DNA extraction from low biomass samples have been proposed to decrease contamination during extraction [13, 14], and a checklist has been recently proposed to guide microbiome studies of samples with low biomass to help minimize and identify contamination [10].

In order to demonstrate “proof-of-life” in low biomass samples, especially those that have historically been considered “sterile,” the use of both microbial culture and fluorescent in situ hybridization (FISH) is typically recommended. While it is likely possible that a microbe of interest, especially anaerobes, may not survive outside of the sample environment, utilizing RNA probes for FISH in a stabilized sample or culturing of specific microbes, such as *Fusobacterium*, should yield supportive evidence as to the existence of metabolically active microbes or polymicrobial communities [15, 16].

## DNA extraction of non-bacterial microbes

The majority of DNA extraction protocols have focused on applications for 16S rRNA gene sequencing or whole genome shotgun metagenomic sequencing in fecal samples. Since the microbes in fecal samples are primarily comprised of bacteria [4], most of the protocols have been optimized for bacterial DNA extraction and may yield biased results for other microbes such as fungi, protists, and viruses. Some studies have evaluated the impact of DNA extraction methods on fungal communities [17, 18], but larger-scale, multi-laboratory studies are needed to evaluate optimal DNA extraction methods for studies of human fungal communities. For studies of viruses, specific DNA extraction methods have been proposed to isolate viral particles and remove contamination by human and microbial cells [19]. However, these methods are focused on solely investigating viruses and therefore remove the other microbes, which indicates a need for a viral DNA extraction protocol to complement DNA extraction for other microbes or a single optimized DNA extraction protocol for all microbes.

## Use of quality control samples during DNA extraction

Due to a lack of standard reference materials for human microbiome studies, quality control samples are not regularly included in microbiome datasets, which can lead to the inability to compare or reproduce results [20, 21]. This can be particularly important when comparing studies utilizing differing DNA extraction methods. Microbiome analyses, at present, can typically use three types of positive reference material: (1) complex environmental samples (e.g., feces from a generous donor) [1, 22], (2) chemostat communities from in vitro microbial model systems [1, 23], and (3) artificial or mock communities [1]. Each type of reference material comes with pros and cons. An advantage of using complex environmental samples is that its composition will closely match the sample of interest which is particularly important for the microbial representation and abundances, in addition to the same environmental matrix. However, the ecosystem is complex with many low-abundance taxa and may be difficult to reproduce if more material is needed later. The advantage of a chemostat is that they approach the complexity of environmental samples and can produce large quantity of reference material, but the supply is still finite as chemostat batches cannot be reproduced at a later time, and some specific taxa may be lost during sample creation. Pure microbial strains and mock communities can be created with known quantity of different bacteria, but they lack the complexity of the environmental samples, are not in the same environmental matrix as the human sample, and only contain culturable microbes. Complex environmental samples or chemostat communities can be used to evaluate reproducibility or consistency of DNA extraction, while artificial or mock community samples can serve as a known ground truth for accuracy. While no reference material is ideal, currently using these reference materials is a good option for standardization within- and between-studies.

Apart from positive controls, negative controls, as described previously in reference to low-biomass samples, are equally critical [1, 20]. Including blanks for each kit or laboratory reagent used during sample handling is key in identification of potential contamination prior to sequencing. At a minimum, both blanks and background environmental contamination controls should be included and analyzed throughout the pipeline. While it is likely not all contamination can be eliminated, identifying and quantifying the contaminants is key to accurate interpretation of results and comparison between studies. Including both positive and negative controls will improve our ability to normalize across users, DNA extraction batches, between experiments, and across laboratories.

## Conclusions and future directions

Ideally in human microbiome studies, we want to extract all or specific microbial DNA found in a sample in order to accurately characterize the microbiota in a sample. Given the lack of a known ground truth for the composition of complex samples, it is still not possible to guarantee validity of a DNA extraction method, but we should at least aim for both consistency and reliability to the greatest extent possible. A great deal of research has been conducted to identify optimal DNA extraction methods for some sample types, particularly fecal samples, but larger-scale studies for optimization of DNA extraction methods for other sample types and non-bacterial microbes are needed. In the meantime, we propose that there are at least three minimal standards that should be followed for human microbiome studies: (1) detailed reporting of DNA extraction methods employed in a study such that another laboratory can easily reproduce all DNA extraction procedures; (2) inclusion and reporting of positive and negative controls in all DNA extraction batches with reporting of coefficients of variation or intraclass correlation coefficients for the positive controls, as well as, information regarding contamination in any blank samples; and (3) utilization of the same DNA extraction protocol across studies for institutions or multi-site studies that plan to pool data in the future (Table 1). Towards this effort, journals have the responsibility to ensure that these aforementioned minimal standards are met prior to publication, as has been implemented in other journals as standard reporting guidelines (e.g., STAR methods in the journal *Cell*). It may never be possible to have a standardized protocol for all human microbiome studies, but scientists, reviewers, and journal editors should strive to improve our current protocols and reporting criteria to increase reliability and consistency between laboratories and studies.

**Table 1:** Recommendations for improved reliability and consistency in human microbiome studies

#1	Collect and process negative controls at each of the following points: during sample collection, DNA extraction, and sequencing
#2	Include at least one or more of the following three types of positive controls in each experiment, depending on study: a complex environmental sample, chemostat community, or mock community
#3	Report in detail the DNA extraction methods followed and sufficient information regarding the results from both positive and negative controls in a manner that allows for peer-review and reproducibility
#4	Utilize the same DNA extraction protocol across studies for multi-institute or multi-site studies
#5	Demonstrate “proof-of-life” beyond sequencing in low-biomass studies using microbial culture and/or FISH
